# Supplementary material for: Haploinsufficiency of RREB1 causes a Noonan-like RASopathy via epigenetic reprogramming of RAS-MAPK pathway genes
Source: Nat Commun. 2020 Sep 16;11:4673. doi: 10.1038/s41467-020-18483-9 (PMC7495420; doi:10.1038/s41467-020-18483-9)

Supplementary Information

Haploinsufficiency of *RREB1* causes a Noonan-like RASopathy via epigenetic reprogramming of RAS-MAPK pathway genes.

Kent et al.

This PDF file includes:

- Supplementary Note 1
- Supplementary Table 1: primer sequences used in the study.
- Supplementary Figures 1-8

Supplementary Note 1

Full case report: The proband is the second child of a healthy non-consanguineous couple. There is no family history of significance. The pregnancy was uncomplicated and he was born at term with a birth weight of 6 lbs and 10 oz and length 19 inches. At birth, bilateral equinus varus deformities of the feet were found and treated with serial casting. An inguinal hernia was repaired at one month of age. Concerns regarding failure to thrive were raised at 9 months of age. He also suffered multiple upper respiratory infections and one episode of pneumonia in childhood, and was found to be IgA deficient. Although there were no delays in his gross motor milestones, he was described as `clumsy'. He was assessed by an ophthalmologist who identified a right exotropia. He required extra assistance in school and an individualized education plan was put in place. At the age of 6, he suffered a grand mal seizure. An EEG identified abnormal generalized spike wave activity. An MRI of the brain showed a focal area of possible gliosis adjacent the left ventricular atrium. His blood karyotype was normal (46,XY). He was commenced on carbamazepine, which was subsequently weaned at the age of 10. However, he sustained three grand mal seizures a year later and a diagnosis of complex partial epilepsy with secondary generalization was made. He was recommenced on anticonvulsant therapy (Gabapentin).

He was first referred to the Clinical Genetics service at age 8 because of proportionate short stature, below the 3rd centile. His father is in the 70th centile and his mother in the 17th centile. Clinical inspection revealed the following findings: He had a tall and broad forehead with widely spaced eyes. His interpupillary length measured 6.8 cm (>98th centile). There was a mild down slant to the palpebral fissures. His ears were posterior rotated and low set. Inspection of his mouth revealed a normal palate and uvula. The facial appearance was reminiscent of a Noonan spectrum disorder. A skeletal survey identified right sided irregularities of the proximal femoral metaphysis, but no other changes to suggest a skeletal dysplasia. A chromosomal array identified a de novo microdeletion of 6p24.3-25.1. He was also found to have incomplete right bundle branch block on ECG. An echocardiogram was unremarkable. He continued to be followed by the Immunology service and was diagnosed with

dysgammaglobulinemia because of his poor ability to maintain an antibody response over time. He was commenced on Amoxicillin prophylaxis.

At 14.5 years of age, his growth parameters were as following: OFC 54.5 cm (<50th centile), weight 41.7 kg, and height 154.2 cm (3-10th centile). In view of his facile gestalt, a diagnosis within the Noonan spectrum disorder was explored further with a targeted Noonan syndrome gene panel and then whole exome sequencing**.** No pathogenic variants in any of the known Noonan syndrome genes were identified.

Supplementary Table 1: primer sequences used in the study.

(A) Primers used for QPCR

Gene	Species	FW	RV
**BACT**	Human	AGGCACCAGGGCGTGAT	GCCCACATAGGAATCCTTCTGAC
**FGFR4**	Human	ACAGCTCTCAGGGACCCAAG	GCTGGAGCTGGGAGTGAG
**HRAS**	Human	CCAGCTTATATTCCGTCATCG	CAGTCGCGCCTGTGAAC
**JUN**	Human	CCCCCAGCGTATCTATATGGAA	GCTGTCCCTCTCCACTGCAA
**LY86**	Human	CACTTTTCAACAGAAAAGCCA	CCACACACGTGGTCTGTAGC
**MAP2K2**	Human	AAGGCCTCCAAGTTGGTCTC	CAAAATGCCCAAGAAGAAGC
**MYC**	Human	CACCGAGTCGTAGTCGAGGT	TTTCGGGTAGTGGAAAACCA
**RREB1**	Human	CCTCTGAAACGTAGGCGATTGT	GGCATCGTGACTCAGTTTCCTC
**SIN3A**	Human	GCCTCAACGAGCACAACCA	GGGCTGTGTTCACCAGGAA
**SSR1**	Human	CTTCAGGTTCACCAGACACA	GGATGAAGATGATGAAGCCG
**Fgfr4**	Mouse	CACCGTGGCTGTGAAGATG	CTTCATCACCTCCATCTCGG
**Gapdh**	Mouse	TGTGTCCGTCGTGGATCTG	GATGCCTGCTTCACCACCTT
**Hras**	Mouse	CCCTTGGGTCAGGCATCTAT	TCTACAGCGGCTGCCAAT
**Jun**	Mouse	GGGACACAGCTTTCACCCTA	GAAAAGTAGCCCCCAACCTC
**Map2k2**	Mouse	GACGGCGAGATCAGCATC	ATGCTGACCTTCCCCAAGAT
**Myh6**	Mouse	CTTCATCCATGGCCAATTCT	GCGCATTGAGTTCAAGAAGA
**Myh7**	Mouse	GAGCCTTGGATTCTCAAACG	GTGGCTCCGAGAAAGGAAG
**Nppa**	Mouse	CAGAATCGACTGCCTTTTCC	GGGGGTAGGATTGACAGGAT
**Nppb**	Mouse	ACCCAGGCAGAGTCAGAAAC	ACAAGATAGACCGGATCGGA
**Rreb1**	Mouse	GGCAGTCAGGCGATTTGGA	AGTGGGTTATCTGAGTGGGTC

(B) Primer used for ChIP

Gene	Species	FW	RV
**ARHGEF2**	Human	GTCTCGGGGACAGGAAGTCT	CTACCCCCTTCCTGAGCTTG
**FGFR4**	Human	GTCGCGGGTACATTCCTC	CTCGAGCCTGCGTGACTC
**HRAS**	Human	GGGGAAAGGCTGGGATCC	GAGTAGGGGAGCTGGGGT
**JUN**	Human	CTCGCAACCTCTGATGGAG	GGCAGCGTACTTGGATTCTC
**MAP2K2**	Human	GAGGAGACGGAGGGATGAAG	GGGGTCTTACGGGTTCTCTC
**MYC**	Human	GTCCGGGGAGGAAAGAGTTA	TGGGAGAAATCAAAGGTGCT
**Fgfr4**	Mouse	GACCACGCCTCTCAGATCA	CAGGAATGTCCCTGCAACTG
**Hras**	Mouse	GAGCAGAAGGCCCTGGTT	GACTCGCTCTGCCTGTGG
**Map2k2**	Mouse	CCTCGCAGAAAAAGATCTCG	GAGGCCGAGTGAAGCTGAC

(C) Primers for genotyping

Gene	Species	FW	RV
**RREB1(a)**	Mouse	TTGGGAGATGGAGAAACTTCACTG	AGTCTACTAATGGCTCAATGGTGC
**RREB1(b)**	Mouse	GAGGAGAAGTCTTCCTACAACTGTC	GTGTTTGAAAGAGAGCGAGAGAAC

Notes: (a) WT and HET alleles 320nt PCR; (b) WT allele 2024nt, HET allele 842nt PCR.

(D) Primers for cloning

Cloning	FW	RV
**BiFC**		
**VC-RREB1**	ACCGAGATCTCTCGAGTGATGACGTCAAGTTCGCCCG	TGGATCCCCGCGGCCGTCACTCCATCCCCAC
**VN-SIN3A**	ACCGAGATCTCTCGAGTGATGAAGCGGCGTTTGGAT	TGGATCCCCGCGGCCGTTAAGGGGCTTTGAATACTGT
**VN-HDAC1**	ACCGAGATCTCTCGAGTGATGGCGCAGACGCA	TGGATCCCCGCGGCCGCTAGGCCAACTTGACCT
**Promoter**		
**FGFR4**	GCGGCTAGCCAGAAGACAGGGGAGCCAG	GCGGAGATCTGGGAGCGAGGAATGTACCC
**HRAS**	GCGGAGATCTCCTACCATTGGCTGCGCG	GCGGCTAGCTTTGGGTTTGAAAGAGCTAGGA
**MAP2K2**	GCGGAGATCTCCCGAAGAAGGCTGACGC	GCGGCTAGCATTGGAAGTATGCCCGGAGT

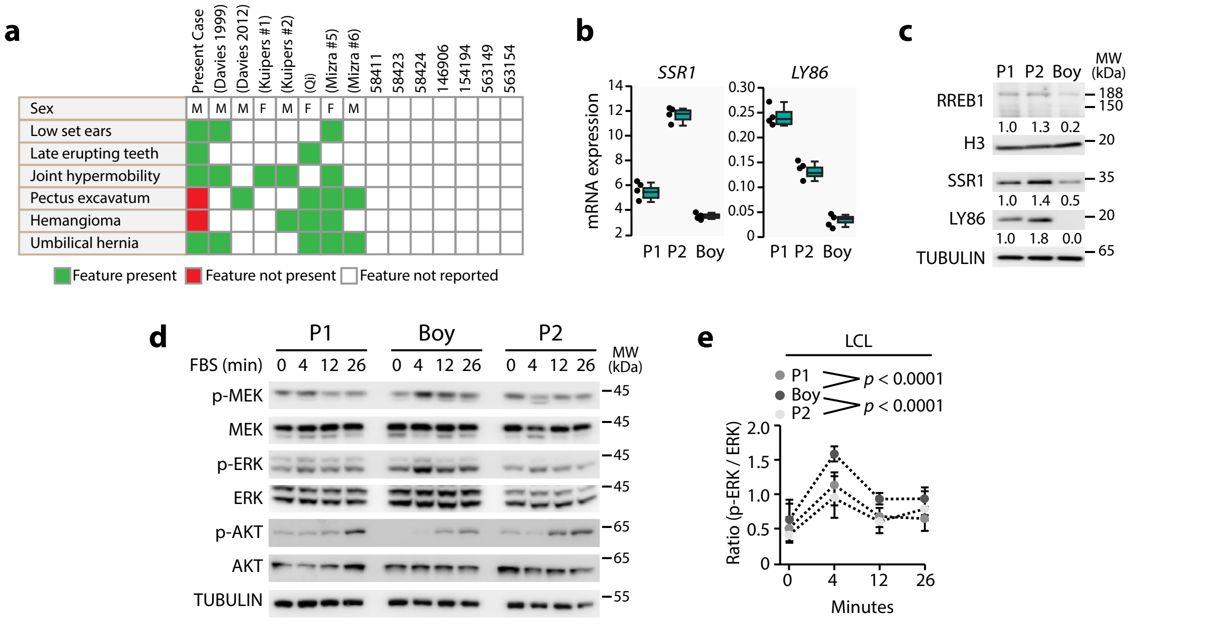

Fig.S1. Haploinsufficiency of *RREB1* in EBV transformed LCL cells. **(a)** Clinical features in addition to those reported in Fig.1 commonly associated with 6p interstitial deletion cases. Cases labeled with numbers refer to unpublished NCBI ClinVar entries. **(b)** mRNA expression (n=6) of *SSR1* and *LY86* in LCLs derived from the proband (Boy) and parents (P1, P2). Box plots indicate the IQR of the data and the central line shows the median. **(c)** Protein expression (n=3) of *RREB1*, *SSR1* and *LY86* in LCLs derived from the proband (Boy) and parents (P1, P2). The western detects the 3 human isoforms of RREB1 at MW 188, 181, and 159 kDa. Tubulin and histone H3 served as loading controls for western. (n=2). **(d)** Analysis of MAPK signaling in LCLs (P1, P2, Boy) following serum deprivation and stimulation with FBS. Shown are the uncut blots displayed in Fig.1. Blots are representative of n=6 experiments. **(e)** Quantification of ERK signaling from westerns in Fig.1 in LCLs derived from the proband (Boy) or parents (P1,P2) following stimulation with FBS. P-values calculated from 4 minute time points. Data average of n=3 independent experiments, p =1.8E-04 (P1 vs. Boy), p =2.9E-04 (P2 vs. Boy) two-tailed student's t-test, error bars presented as mean values +/- SD.

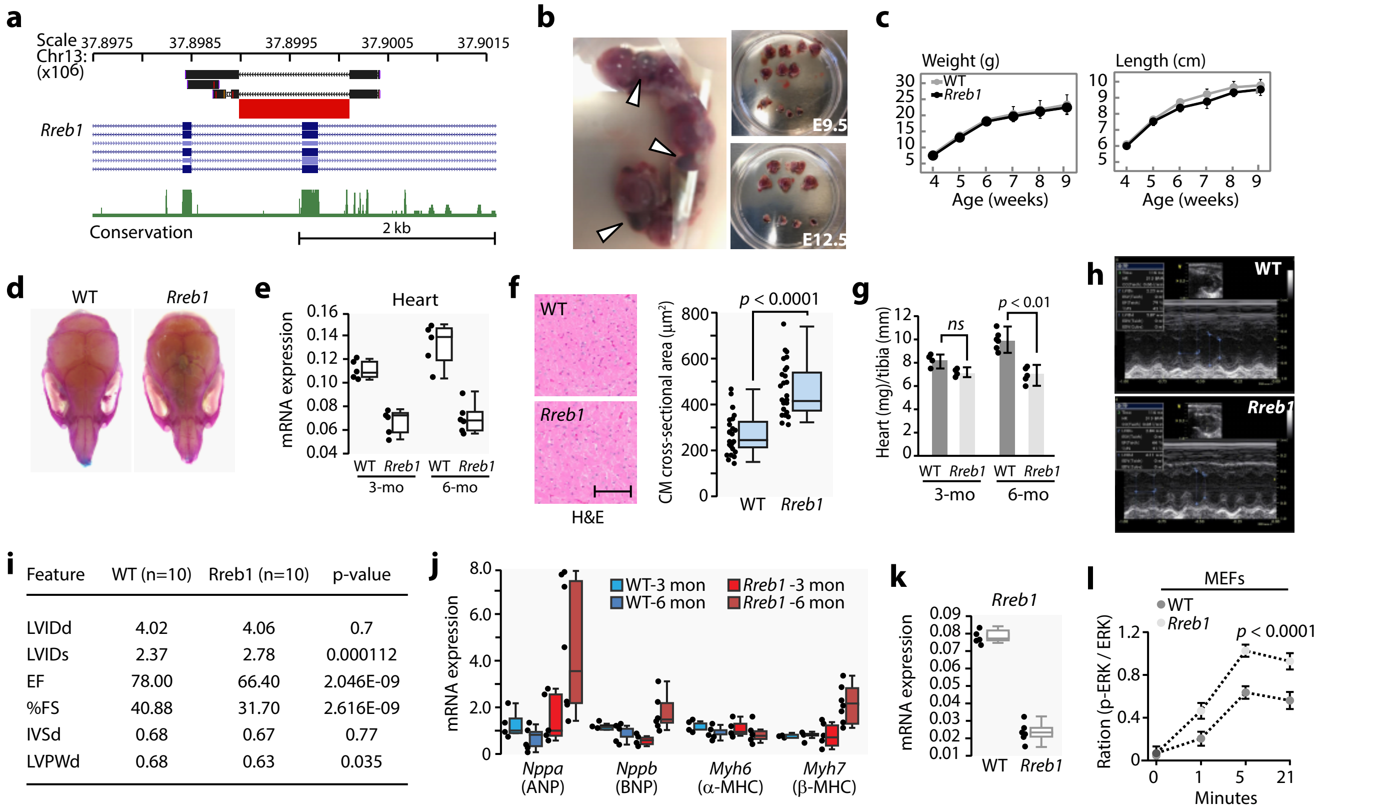

Feature	WT (n=10)	Rreb1 (n=10)	p-value
LVIDd	4.02	4.06	0.7
LVIDs	2.37	2.78	0.000112
EF	78.00	66.40	2.046E-09
%FS	40.88	31.70	2.616E-09
IVSd	0.68	0.67	0.77
LVPWd	0.68	0.63	0.035

Fig.S2. **Features of *Rreb1* hemizygosity in mice. (a)** Expanded genomic region around the murine *Rreb1* gene showing the CRISPR-Cas9 excision boundaries. The sequencing alignment of PCR derived from a representative *Rreb1+/-* founder mouse is shown (black boxes). The deleted region is shown (red box). **(b)** Uterus taken at E9.5 stage with 11 implantation sites including 3 small blood filled sites (arrow-heads). Dissected uterus from E9.5 and a second uterus at E12.5 reveal 12 embryos and 8 empty implantation sites. Genotypes of the embryos were determined (WT=5, HET=7, Null=0). **(c)** Weight and length of wild type and *Rreb1+/-* littermates measured at 4-9 weeks of age. (n=3 WT, n=6 HET). Error bars presented as mean values +/- SD. **(d)** Representative skulls from WT and *Rreb1+/-* male mice stained with alizarin red. **(e)** *Rreb1* mRNA expression in WT and *Rreb1+/-* 3 and 6 month hearts. Normalized to *Acta2* (n=5). Box plots indicate the IQR of the data and the central line shows the median.**(f)** H&E staining of LV cardiac sections from 3 month WT and *Rreb1+/-* hearts. Scale bar 100μm. Quantification of cardiomyocyte (CM) cross-sectional area from 2 hearts in each group measuring12 CM per heart indicated in the box plots. Box plots indicate the IQR of the data and the central line shows the median, p =4.7E-08 two-tailed student's t-test. **(g)** Heart weight/tibia length ratios from 3 and 6 month WT and *Rreb1+/-* mice. (n=4 for 3 month hearts, n=6 for 6 month hearts; mg, milligram; mm, millimeter). Error bars presented as mean values +/- SD, p =2.6E-05 two-tailed student's t-test, ns=not significant. **(h)** Representative echocardiography traces obtained from 6 month WT and *Rreb1+/-* mice. **(i)** Summary of echocardiography data from 6 month WT and *Rreb1+/-* mice (n=10, p-values calculated with two-tailed student's t-test). **(j)** mRNA expression of hypertrophic marker genes from 3 month (n=5) and 6 month (n=8) WT and *Rreb1+/-* hearts. Atrial natriuretic factor, ANF; brain natriuretic factor, BNP; myosin heavy chain, MHC. Box plots indicate the IQR of the data and the central line shows the median. **(k)** *Rreb1* mRNA expression from WT and *Rreb1+/-* MEFs. mRNA normalized to *Acta2* (n=6). Box plots indicate the IQR of the data and the central line shows the median. **(l)** Quantification of ERK signaling in MEFs examined in Fig.2. The ratio of p-Erk and total Erk signal from western blot plotted as a function of time. p =5.9E-05 two-tailed student's t-test calculated from 5 minute time points (n=6, error bars presented as mean values +/- SD).

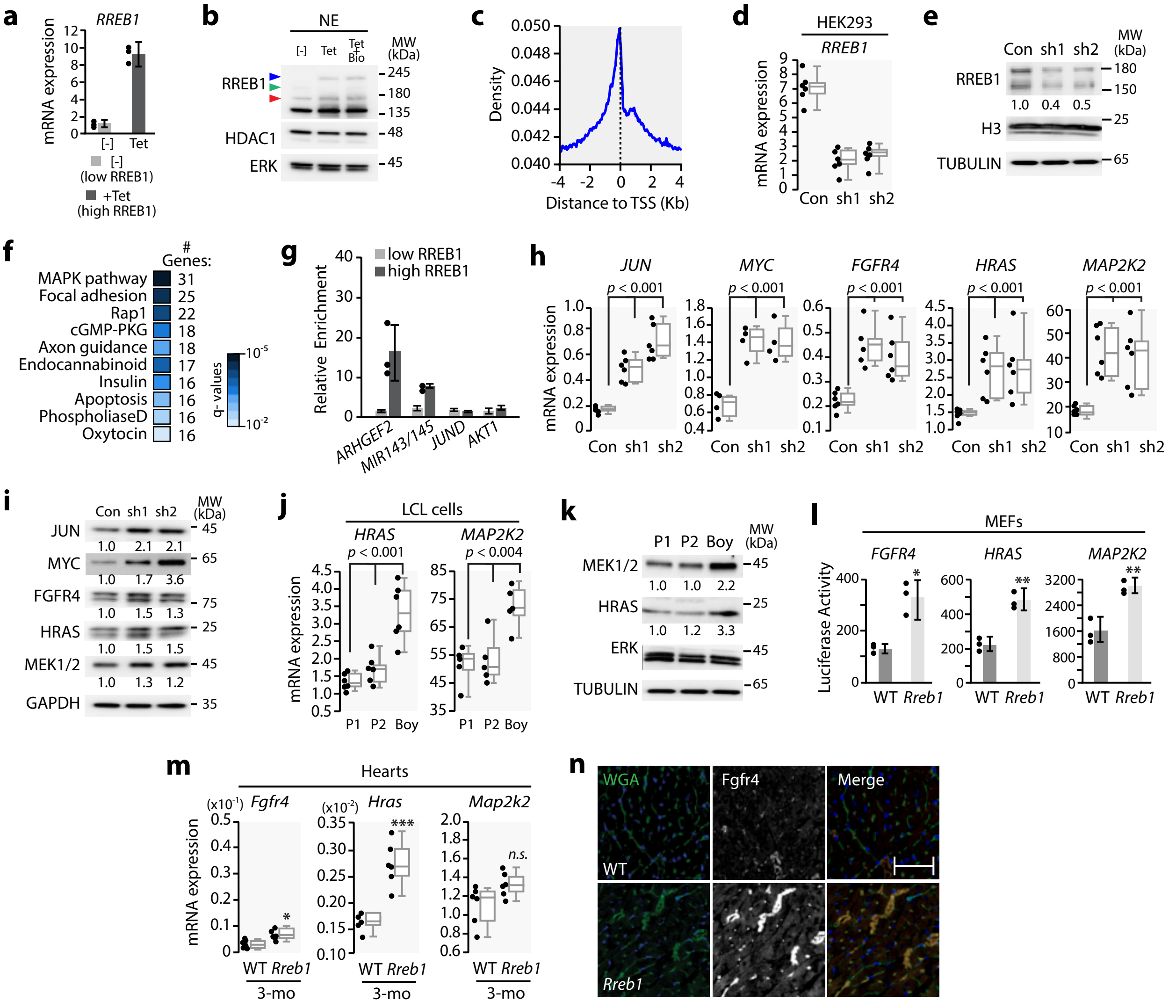

Fig.S3. RREB1 is a transcriptional regulator of MAPK pathway genes. (a) QPCR analysis of *RREB1* mRNA expression in HEK293 cells with a Tetracycline inducible *RREB1* transgene. Cells were treated with DMSO [-] or tet (+Tet) to activate *RREB1* expression. (n=3, error bars presented as mean values +/- SD). **(b)** Western blot analysis of *RREB1* transgene expression following tet activation. Cells treated with tet +bio (Biotin) activate the BirA used for BioID. Flag immunoprecipitation isolates flag-tagged RREB1 used for ChIP experiments. RREB1 in nuclear extract (NE) indicated relative mobility of Flag-BirA-RREB1 (blue arrow), endogenous RREB1 (181/188 MW isoforms green arrow and 159 MW isoform red arrow). HDAC1 and ERK served as controls. (n=2). **(c)** Distribution plot of RREB1 binding around the TSS of target genes as determined by ChIP-seq. **(d)** *RREB1* mRNA expression and **(e)** protein expression in HEK293 cells expressing control shRNA (Con) or one of two shRNA (sh1, sh2) targeting *RREB1*. mRNA expression normalized to *ACTB* (n=6). Box plots indicate the IQR of the data and the central line shows the median. Protein expression is from nuclear lysates. Tubulin and H3 served as loading controls. **(f)** Enrichment of significant signaling pathways ($q < 0.05$) following analysis of differentially expressed genes using the KEGG database. **(g)** ChIP analysis o the indicated TSS amplicons at the indicated genes in flag-tagged RREB1 ChIP from HEK293 cells treated with DMSO (low RREB1) or tetracycline to induce *RREB1* expression (high RREB1). Enrichment is relative to IgG antibody. Data represented as average of 3 independent experiments, error bars presented as mean values +/- SD. **(h)** Expression of the indicated mRNAs and **(i)** proteins in HEK293 cells expressing control shRNA (Con) or shRNA (sh1 or sh2) targeting *RREB1* (n=4 MYC, n=5 JUN, FGFR4, n=6 HRAS, MAP2K2; indicated *p* -values based on two-tailed student's t-test to compare sh1 or sh2 vs. shCon). Box plots indicate the IQR of the data and the central line shows the median. GAPDH served as a loading control. **(j)** *HRAS* and *MAP2K2* mRNA expression (n=5; p =0.0002 (P1 vs. Boy-*HRAS*), p =0.002 (P2 vs. Boy-*HRAS*), p =0.001 (P1 vs. Boy-*MAP2K2*), p =0.001 (P2 vs. Boy-*MAP2K2*) based on two-tailed student's t-test) and **(k)** protein expression (n=3) in LCLs derived from the proband (Boy) and parents (P1, P2). mRNA expression normalized to *ACTB*. Box plots indicate the IQR of the data and the central line shows the median. ERK and tubulin served as loading controls for western blots. **(l)** Promoter activation assay of human *FGFR4*, *HRAS*, and *MAP2K2* promoters expressed in WT and *Rreb+/-* MEFs (n=3; *p<0.01, **p<0.001 two-tailed student's t-test, error bars presented as mean values +/- SD). **(m)** Expression of the indicated genes in WT and *Rreb1+/-* hearts obtained from 3 month mice (n=5; *p<0.01, ***p<0.0001 two-tailed student's t-test, n.s.=not significant). Box plots indicate the IQR of the data and the central line shows the median. **(n)** Fgfr4 in LV sections from WT and *Rreb1+/-* hearts. Wheat germ agglutinin (WGA) marks the CM membranes. Scale bar 100 μm. n=3 hearts for each WT and HET.

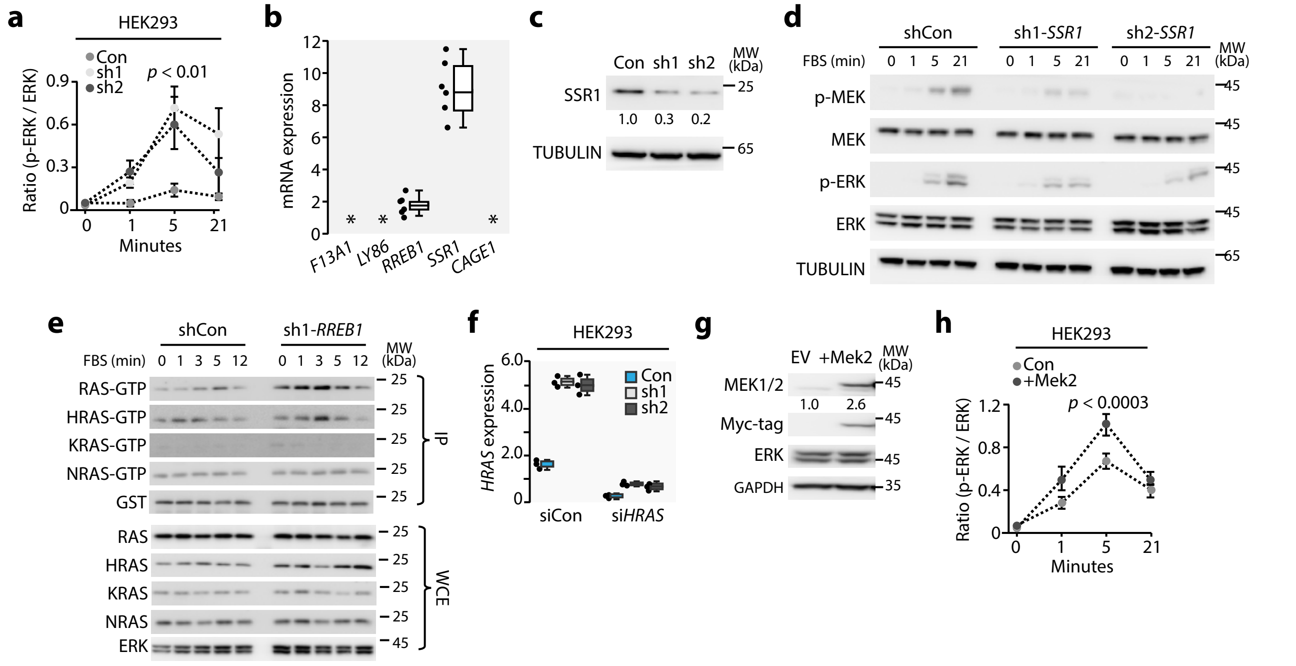

Fig.S4. RREB1 regulates sensitization of FGFR4-HRAS-MAPK signaling. (a) Quantification of ERK activation in HEK293 cells expressing shCon or sh1, sh2-*RREB1* examined in Fig.4. The ratio of p-ERK and total ERK is plotted as a function of time. p =0.01 (Con vs. sh1), p =0.002 (Con vs. sh2), value two-tailed student's t-test calculated from 5 minute time points. Data average of n=6 independent experiments, error bars presented as mean values +/- SD. **(b)** Expression of the indicated genes indentified in the microdeletion case and analzyed in HEK293 cells. Data represented as box plots from 3 independent measurements. Box plots indicate the IQR of the data and the central line shows the median. Genes marked with an asterisk were undetected. **(c)** SSR1 protein expression in HEK293 cells expressing control shRNA (Con) or one of two shRNA (sh1, sh2) targeting *SSR1*. Tubulin served as a loading control. (n=3). **(d)** RAS-pathway activity in HEK293 cells expressing control shRNA (shCon) or shRNA (sh1, sh2) targeting SSR1 following stimulation with FBS at the indicated times (n=5). **(e)** Western blot analysis of RAS activation in HEK293 cells expressing shCon or sh1 targeting *RREB1* (n=3). RAS isoforms were detected by immunoblotting with the indicated RAS antibodies. The GST probed blot detects GST-RBD used for affinity-purification of activated RAS-GTP (IP blots). ERK served as a protein loading control for whole cell extract (WCE) blots. **(f)** *HRAS* mRNA expression in HEK293 cells stably expressing control (shCon) or shRNA (sh1, sh2) targeting *RREB1* 72 hours post transfection with control (siCon) or siRNA targeting *HRAS* (si*HRAS*). mRNA expression was normalized to *ACTB* (n=3). Box plots indicate the IQR of the data and the central line shows the median. **(g)** Western blot analysis of MEK2 over expression in HEK293 cells expressing empty vector control (EV) or Myc-tagged MEK2 (+Mek2, 0.1 μg plasmid). The MEK blot was probed with the MEK1/2 antibody. ERK and GAPDH served as loading controls. Quantification of MEK2 expression is indicated (n=4). **(h)** Quantification of p-ERK signaling in HEK293 cells expressing empty vector control (EV) or Myc-tagged MEK2 following stimulation with FBS examined in Fig.4 (n=4). p =2.6E-04 (Con vs. Mek2) value two-tailed student's t-test calculated from 5 minute time points, error bars presented as mean values +/- SD.

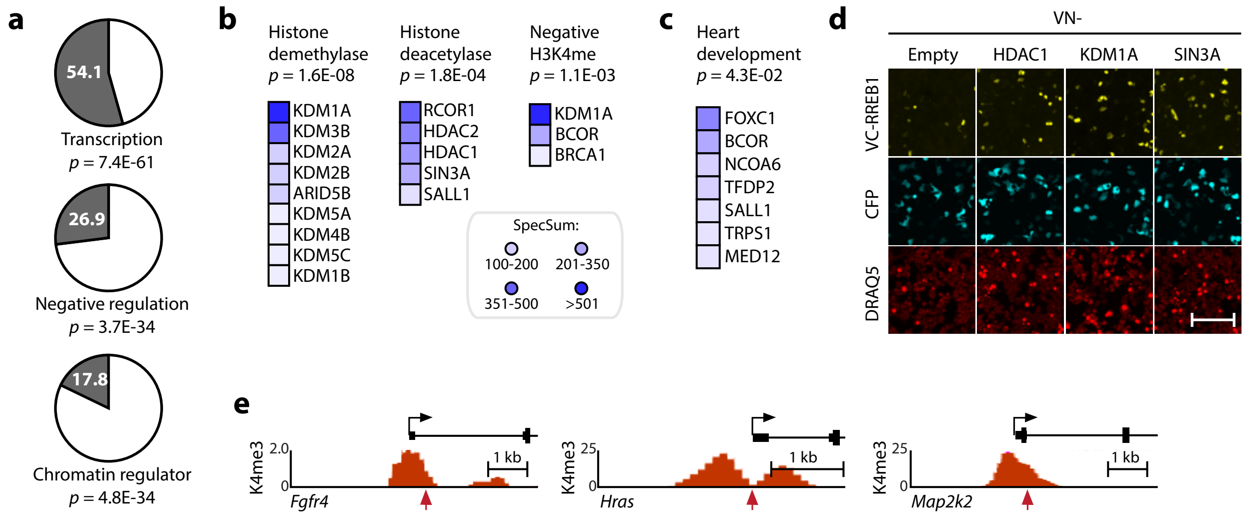

Fig.S5. Analysis and validation of the RREB1-BioiD. **(a)** Broad characteristics of the RREB1 interactome identified by DAVID analysis. Indicated *p*-values based on one-tail Fisher exact probability value. **(b)** RREB1 interactors identified by Gene Ontology (GO) and UniprotKB keyword analysis with the indicated functions provided by DAVID. Box color represents spectral sum counts (SpecSum) as indicated in Fig.3a. Indicated *p*-values based on one-tail Fisher exact probability value. **(c)** RREB1 interactors associated with heart development identified by DAVID analysis. Indicated *p*-values based on one-tail Fisher exact probability value. **(d)** BiFC validation of RREB1-protein interaction. Venus-C-terminal-RREB1 (VC-RREB1) was co-transfected with the indicated Venus-N-terminal (VN-) constructs. CFP expression was used as a transfection control and DRAQ5 was used to mark the nucleus. Images are representative of n=3 replicated experiments. Scale bar 10 μm. **(e)** Genomic regions around the TSS of murine *Fgfr4*, *Hras*, and *Map2k2* genes showing H3K4me3 binding observed in 8-week mouse hearts (ENCODE, LICR, 8-week adult heart). Arrows point to a putative murine RRE.

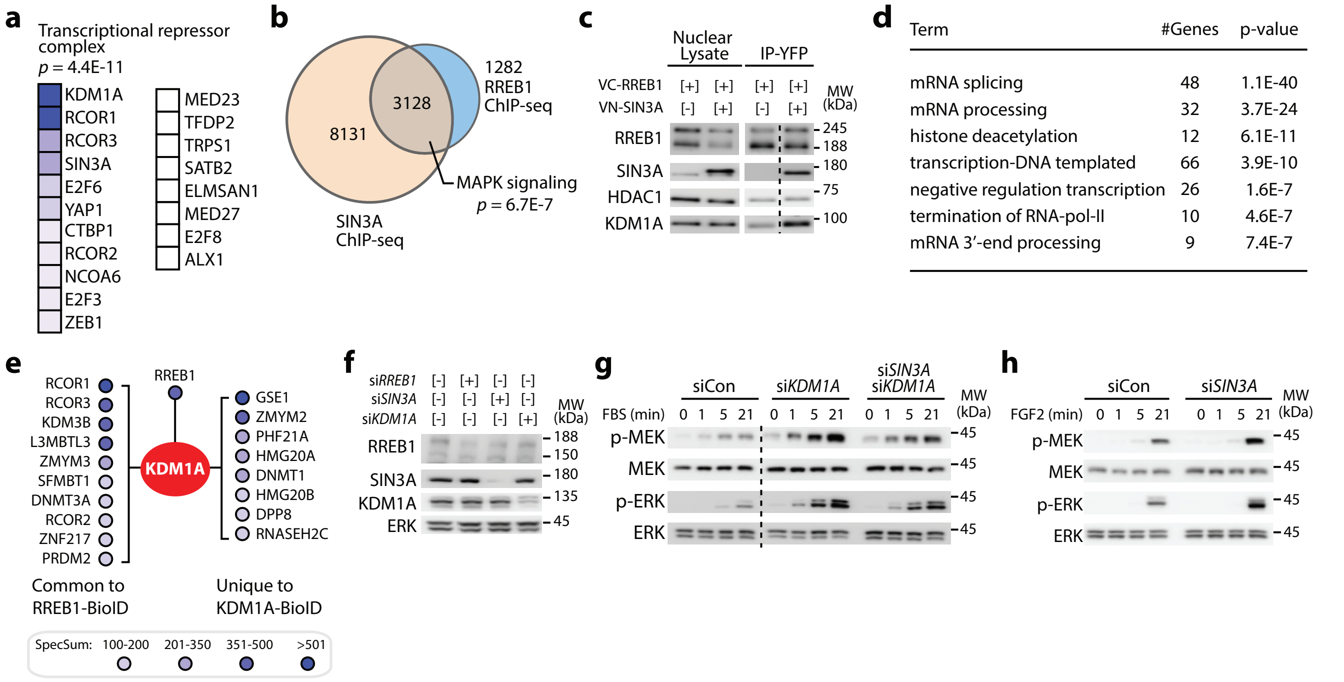

Fig.S6. Additional features of the RREB1-SIN3A-KDM1A complex. **(a)** RREB1 interactors identified by RREB1 BioID associated with GO molecular function term defined as transcriptional repressor complex. Box color represents spectral sum counts (SpecSum) as indicated in Fig.5. Indicated *p*-value based on one-tail Fisher exact probability value. **(b)** venn diagram of the peak overlap of SIN3A ChIP-seq and RREB1 ChIP-seq datasets. The RREB1 ChIP-seq genes are those enriched for an RRE motif identified by the transfac matrix database (v7.0) annotated in human, mouse, rat alignment. KEGG analysis of genes in the overlap revealed MAPK signaling signature. Indicated *p*-value based on one-tail Fisher exact probability value. **(c)** validation of VC- and VN- overexpression constructs used for YFP pull down experiment. Western blot analysis of expression of Venus-C-terminal-RREB1 (VC-RREB1) and Venus-N-terminal (VN-) SIN3A constructs as indicated. (n=2). **(d)** Select terms identified by GO biological processes for components of the RREB1-SIN3A complex. **(e)** KDM1A interactors identified by KDM1A-BioID (1% FDR). Dot color is representative of spectral sum counts (SpecSum) as indicated in the scale. Indicated *p*-values based on one-tail Fisher exact probability value. **(f)** Western blot of indicated proteins in lysates from HEK293 cells treated with control siRNA (-) or siRNA (+) targeting *RREB1*, *SIN3A* or *KDM1A* as indicated. ERK served as loading control. (n=3). **(g)** MAPK signaling in HEK293 cells expressing siRNA targeting *KDM1A* or *SIN3A* as indicated following stimualtion with FBS (n=3). **(h)** MAPK signaling in HEK293 cells expressing siRNA control (siCon) or siRNA targeting *SIN3A* following stimulation with FGF2 (n=3).

Fig.S7. Uncropped blots Figures 1-6.

Figure 1f

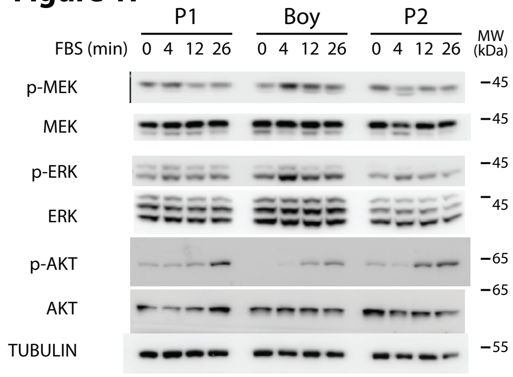

Figure 2j

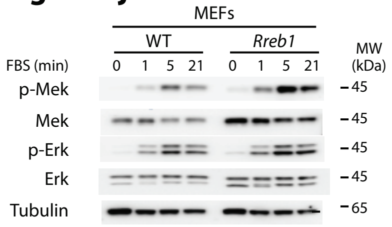

Figure 3j

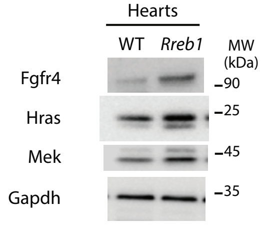

Figure 4a

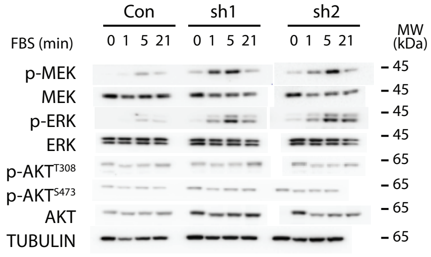

Figure 4d

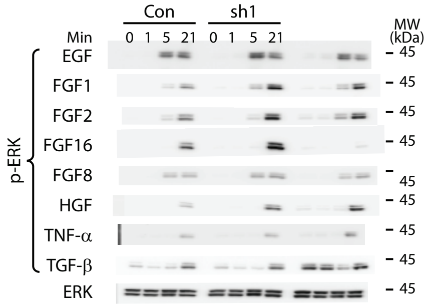

Figure 4g

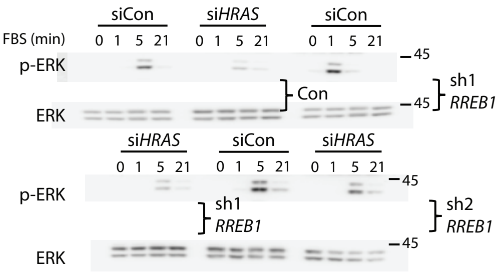

Figure 4h

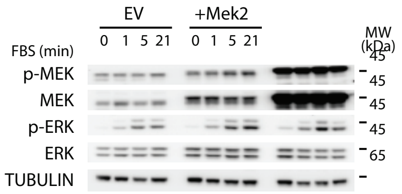

Figure 5b

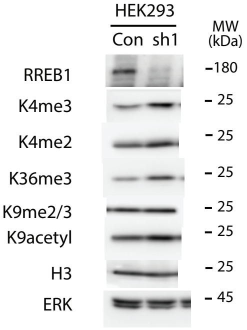

Figure 5c

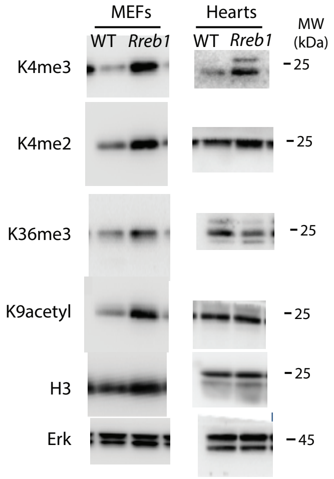

Figure 6c

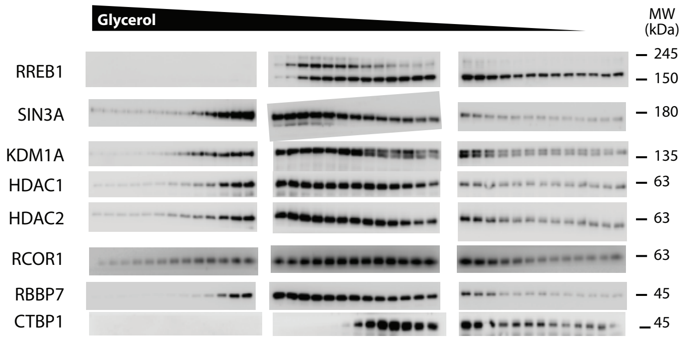

Fig.S8. Uncropped blots Supplementary Figures 1-6.

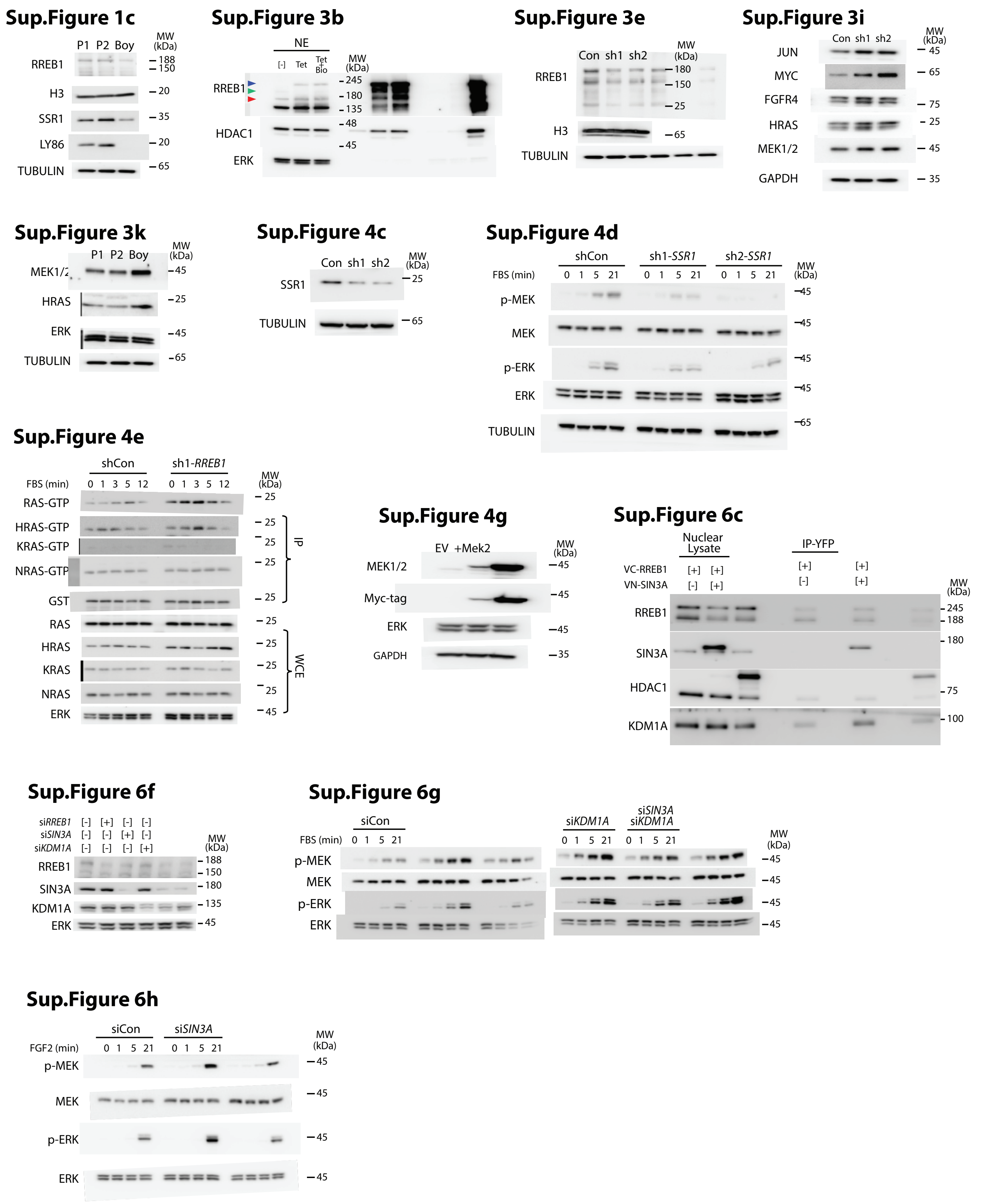

Supplement: Supplementary file 1 — Supplementary Information [file 41467_2020_18483_MOESM1_ESM.pdf]
